# Advances in Human Pathogen Control—A 21st Century Challenge

**DOI:** 10.3390/vaccines11091449

**Published:** 2023-09-02

**Authors:** Jorge H. Leitão, Joana R. Feliciano, Nitin Amdare

**Affiliations:** 1 Department of Bioengineering (DBE), Institute for Bioengineering and Biosciences (iBB), The Associate Laboratory Institute for Health and Bioeconomy (i4HB), Instituto Superior Técnico (IST), Universidade de Lisboa, 1049-001 Lisbon, Portugal; joana.feliciano@tecnico.ulisboa.pt; 2 Department of Microbiology and Immunology, Albert Einstein College of Medicine, Bronx, NY 10461, USA; nitin.amdare@einsteinmed.edu

The emergence of new pathogens, coupled with the reemergence of old pathogens and the steep worldwide increase in multiple resistances to available antimicrobials, poses major challenges to human health at the global scale. Adequate responses to these challenges require a holistic approach, integrating various disciplines, such as pathogen biology, omics, and bioinformatics, to unveil druggable targets and for the development of novel antimicrobials and immune-based therapies to control these pathogens. This Special Issue gathered contributions ranging from basic biology and microbiology, to biochemistry, genetics and genomics, multi-omics, and bioinformatics, in order to identify targets of potential interest for the development of novel antimicrobials, innovative immune-based therapies to control pathogens, and the combined use of already existing drugs with newly potentiators of their antimicrobial activity.

The SARS-CoV-2 pandemic that emerged by the end of 2019 led several research groups to re-direct their activities towards focusing on the study of the biology of the virus, the identification of druggable targets, and the development of therapies. As a consequence, we assisted the unprecedented fast development and approval of new vaccines that undoubtedly saved millions of lives worldwide. Among the 15 papers published in this Special Issue, SARS-CoV-2 is the theme of three of them. López-Cortés and co-authors [1] elegantly reviewed knowledge about the proteins ANPEP/CD13, DPP IV/CD26, and ACE2, human receptors used by coronaviruses. These receptors are moonlighting enzymes involved in several physiological processes, and are expressed in various tissues and distinct cell types. The work reviewed the commonalities between the three enzymes, and their physiological functions, showing that blocking any of those enzymes might result in systemic deregulations and multi-organ failures [1].

The severity of the disease caused by SARS-CoV-2 was addressed by Alsrhani and colleagues [2]. In their work, these authors addressed the role played by D-dimer in patients with COVID-19, and investigated its association with the progression and severity of the disease in a study involving two Saudi medical centers [2]. The authors quantified plasma D-dimer levels in the samples collected from 148 COVID-19 patients, and found that the plasma D-dimer concentrations were significantly elevated in COVID-19 patients compared to normal, uninfected controls. Plasma D-dimer concentrations were significantly increased in patients with mild infection to moderate disease, but the highest levels were detected in patients with severe COVID-19 disease [2]. These results led the authors to conclude that D-dimer can be used as a biomarker for both the detection of COVID-19 infection and the for severity of the disease [2].

The transmission of SARS-CoV-2 particles through air was investigated by Garg and co-authors [3]. These authors pointed out the need to develop mechanisms to effectively reduce the viral load in the air, particularly in closed and crowded places such as hospitals and public transport. The virus inactivation kinetics by UVC were investigated to identify conditions leading to maximal virus inactivation. The authors report the design of experimental UVC-based devices for air sanitization through heating, ventilating, and air conditioning (HVAC) systems in closed spaces. The authors concluded that UVC radiation could reduce the risk of infection by up to 90% [3].

HIV remains a major pathogenic virus, estimated by the World Health Organization (WHO) to have claimed 40 million lives thus far [4]. The WHO estimates that 39 million people live presently with the virus, with two thirds living in Africa [4]. The diagnosis of the infection at an early stage of HIV infection (acute HIV infection, AHI), plays an important role in immune system failure and HIV transmission. However, early detection of AHI patients is often missed due to non-specific symptoms. To facilitate the identification of AHI patients, Shi and co-authors [5] present a study involving 61 AHI patients in a Southwest China hospital, in which a novel HIV screening assay called Elecsys^®^ HIV Duo was used. The authors highlight the importance of the correct diagnosis of HIV at the acute phase [5].

Raj et al. [6] present a study on the use of a soluble hemagglutinin (HA) glycoprotein-based protein subunit vaccine to combat the influenza virus, the causative agent of flu. Despite the availability of flu vaccines for almost 50 years, influenza viruses remain a potential pandemic threat. The authors expressed and purified a recombinant soluble trimeric HA protein from the highly virulent Inf A/Guangdong-Maonan/SWL1536/2019 virus, and show its efficacy in protecting BALB/c mice against a lethal dose of the virus after subcutaneous administration [6]. These promising results led the authors to propose trimeric HA as a suitable vaccine candidate against the influenza virus [6].

*Streptococcus agalactiae* is a commensal bacterial species, commonly found in the human gastrointestinal and urogenital tracts. The bacterium can cause severe infections, particularly among pregnant women and newborns. A major concern is the ability of the bacterium to acquire resistance to multiple antibiotics, often leading to treatment failure. Djuikoue and co-authors [7] report a prospective cross-sectional study in a hospital setting in Cameroon, by collecting and analyzing vaginal and urine swabs collected from 163 women, and the results obtained showed that non-pregnant and pregnant women were, respectively, 37% and 59.7% colonized with *S. agalactiae. *The authors highlight the importance of monitoring this bacterium antimicrobial resistance in pregnant women and newborns [7].

The evolving resistance to multiple antibiotics is a major concern, and the routes of resistance spreading in the environment are barely understood. Mutuku and co-authors [8] evaluated the prevalence of β-lactam resistance in clinically important enteric bacteria of the species *Klebsiella* sp., *Escherichia coli*, and *Enterobacter* sp. found in wastewater, including hospital effluents, municipal wastewater, and wastewater treatment plants. A multidrug resistance phenotype was found in 72% of *E. coli* isolates, 70% of *Klebsiella* species isolates, and 40% of *Enterobacter* and *Citrobacter* species. ESBL genes were found in *E. coli *and *Klebsiella* species. Genes involved in antibiotic resistance, encoding for aminoglycoside modifying enzymes, adenylyltransferases, phosphotransferases, acetyltransferases, sulfonamide/trimethoprim resistant dihydropteroate synthase, dihydrofolate reductase, and quinolone resistance were also identified [8]. The study is an important contribution in the design of effective strategies to minimize the environmental spread of resistant genes.

Infections caused by the protozoa *Plasmodium falciparum* and *Trypanosoma cruzi*, the causative agents of malaria and Chagas disease, were also the subject of two papers. Carvalho and co-authors [9] investigated the association between pyruvate kinase deficiency and resistance to malaria. The authors used atomic force microscopy and other experimental approaches to investigate modifications of red blood cell morphology, membranes, and biomechanical properties on in vitro cultures of *Plasmodium falciparum* treated with 2,3-diphosphoglycerate [9]. The results indicate that 2,3-diphosphoglycerate has only a mild effect on red blood cells when compared with the presence of the parasite on the host cell. The authors anticipate that, in the future, diphosphoglycerate may be exploited to design a new antimalarial tool [9]. Banga and co-authors work call attention to the urgent need to identify pathways and targets to develop novel drugs against *Trypanosoma cruzi* [10]. The authors focused their attention on cyclic AMP (cAMP)-specific phosphodiesterases (PDEs), as cAMP is a key regulator of mammalian cell proliferation and differentiation, playing a role in *T. cruzi* growth. Several xanthine analogs were screened against trypomastigote and amastigote growth, and the GVK14 xanthine analog was identified and shown to inhibit the studied strains of amastigotes in host cells [10]. The authors conclude that xanthine analogs capable of inhibiting *T. cruzi* PDE should be regarded as novel alternative therapeutics to combat Chagas disease [10].

Human lymphatic filaria is another example of a disease caused by an eukaryotic parasite. Bhoj and co-authors [11] reviewed the strategies used by these worm pathogen strategies to secure their long-term survival in the host. The authors also highlight the importance of this knowledge in understanding the biology of these parasitic and inflammatory diseases. The knowledge of the intricate network of host immune–parasite interactions is expected to lead to the development of novel and effective immune-therapeutic options [11].

The intestinal disease cryptosporidiosis affects several hosts, including animals and humans. Drug treatment is the only available treatment for the disease, as no vaccines are available against it. Human cryptosporidiosis can be treated with the only approved drug, namely nitazoxanide (NTZ) [12]. Since the efficacy of this drug is limited among people with conditions such as compromised immune systems or malnourishment, Nguyen-Ho-Bao and co-authors [13] studied the potential of the cell-penetrating peptide octaarginine to increase the uptake of NTZ. The authors have synthetically attached octaarginine to NTZ and used the synthesized compound in *Cryptosporidium parvum* growth inhibition studies in vitro. The authors present results showing the benefits of this modification in the activity of NTZ. Nevertheless, further work is necessary to put this strategy for cryptosporidiosis treatment into practice [13].

Several infections are not due to a single pathogen alone. This is the case with infections caused by *Staphylococcus aureus* and *Candida albicans*, which include periodontitis, cystic fibrosis, denture stomatitis, urinary tract infections, burn wound infections, and infections associated with invasive medical devices such as venous catheters [14]. Mahmoud and co-authors [14] studied the antimicrobial activity of the monoterpene bicyclic derivative myrtenol in pure and mixed cultures of both organisms, as well as in combination with antibacterial, antifungal, and disinfectant compounds. The results obtained led the authors to conclude that myrtenol potentiates the antimicrobial and anti-biofilm activity of the examined compounds against mono- and dual-species cultures of *S. aureus* and *C. albicans* [14].

The search for natural and synthetic compounds with antimicrobial activity has attracted many research groups in recent years, due to the worldwide emergence of pathogens resistant to commercially available antimicrobials and the reduced number of new antimicrobials coming onto the market [15]. Le Gal and co-authors report the analysis of sixteen structurally related monoanionic gold (III) bis (dithiolene/diselenolene) complexes, differing in the heteroatom linked to the gold atom (AuS for dithiolene, AuSe for diselenolene), the type of chemical substituents on the nitrogen atom of the thiazoline ring, the nature of the exocyclic atom or group of atoms, and the counter-ion used to produce the salts of the complexes [16]. The anticancer, antimicrobial, and anti-HIV activities of the complexes were assessed. Most of the studied complexes exhibited anticancer activities against Cisplatin-sensitive and Cisplatin-resistant ovarian cancer cells [16]. Several of the studied complexes were also found to be active against the *Plasmodium berghei* parasite in its liver stage. The authors highlight the potential of the studied complexes as future drug candidates for therapeutical applications [16].

Whitehead and co-authors [17] examined the extent of microbial transfer from washing machines to clothes through the laundering process. Although washing machines effectively remove dirt from cloth, modern cold-water machine-washing practices are not designed for microbial eradication. The authors used bacterial 16S rRNA and fungal ITS sequencing techniques to identify bacteria and fungi transferred to clothes during the washing process, and enumerated the total viable organisms by plating techniques [17]. The authors report the recovery of various opportunistic human bacterial pathogens, such as *Enterococcus faecium*, *Staphylococcus aureus*, *Klebsiella pneumoniae*, *Acinetobacter baumannii*, *Pseudomonas aeruginosa*, and *Enterobacter* spp. [17]. The authors conclude that public washing machines are a possible source of contamination of clothes by both non-pathogenic and pathogenic microorganisms [17].

The introduction of powerful techniques for faster and more accurate identification of pathogens in the clinical microbiology laboratory is a critical need. An example is the successful use of matrix-assisted laser desorption ionization time-of-flight mass spectrometry (MALDI-TOF MS) in clinical microbiology laboratories, which began a decade ago. The advantages of the technique over conventional methods include its easy use, speed, accuracy, and low cost [18]. Elbehiry and co-authors review the historical developments of the technique, and the usual workflow in the identification of microbial pathogens, and anticipate that MALDI-TOF MS will play a significant role in advancing clinical microbiology [18].

Altogether, this collection of papers illustrates various advances in the knowledge of the biology of various human pathogens and how this knowledge is being exploited, using state-of-the-art methodologies to design novel strategies, conceive new therapeutical approaches, and design new drugs to combat human pathogens.
